# Influence of conversation technique seminars on the doctoral therapeutic attitude in doctor–patient communication

**DOI:** 10.1007/s00508-016-1023-8

**Published:** 2016-06-22

**Authors:** Sandra Drdla, Henriette Löffler-Stastka

**Affiliations:** Klinik für Psychoanalyse und Psychotherapie, Medizinische Universität Wien, Währinger Gürtel 18–20, 1090 Vienna, Austria

**Keywords:** Communication, Physician–patient relations, Medical students, Attitude, Education

## Abstract

This study investigates medical students’ therapeutic attitude before and after communication skills training seminars with simulated patients. The aim was to find out whether the therapeutic attitude of medical students is trainable and whether there is a difference in therapeutic attitude before and after the communication skills training with standardized patients. The participating groups are medical students in their 4th year. The collected parameter is the therapeutic attitude on the basis of the therapeutic attitude questionnaire. The questionnaires are filled out at two different points in time, which are the following: once before and the second time after the communication skills training. The results of this study indicate that therapeutic attitude is trainable. Further studies in the area of communication skills training in medical students are needed to emphasise these results.

## Introduction

A good physician–patient relationship is essential for the patient’s health [1–5]. Accordingly, the factor “therapeutic relationship” can influence the success of the therapy with a variance of 30 % [6]. A hypothesis-based, well-structured, patient-centred anamnesis enables an earlier and more accurate diagnosis and eventually an earlier and more precisely targeted therapy [3, 7]. Empathic behaviour towards the patient leads to higher patient’s satisfaction, better and timelier diagnosis, improved adherence to treatment, fewer complaints and a more effective coping with the illness [3, 8–10]. Young et al. showed that furthermore the perceived competence of the physician by the patient is essential for his/her outcome [7, 11]. The patients’ satisfaction correlates with the receipt of an adequate explanation, fulfilment of expectations and duration of the conversation [12].

All these aspects should be taught in medical universities to prepare medical students to the clinic. Aim of this study was to find out whether the therapeutic attitude in medical students is trainable and whether there is a difference in medical students’ therapeutic attitude before and after the communication skills training.

The communication skills training “Physician–patient communication with simulated patients” (Gesprächsführungsseminare C) takes place in the fourth year, training focuses on challenges in communicating with patients in a challenging setting and on the affective involvement in doctor–patient relationships. Front lectures, textbook and a mandatory e‑learning program [13–16] provide background. Afterwards group seminars with simulated patient contact require students to successfully take mental states. The main aim is to learn to integrate theoretical knowledge in the communication and to manage the special setting with psychiatric patients.

During the second and third year students use roleplaying situations in group seminars, called Ärztliche Gesprächsführung A und B (ÄGF A and ÄGF B), to learn and practice general medical history taking. The goal is to learn to take a complete and well-structured anamnesis in an empathic and patient-centred way.

Overall, the Viennese Medical Curriculum (MCV) [17] lays a focus on integrative, horizontal and problem-oriented learning, the ability for conducting interviews besides clinical–practical skills should be gained early in the curriculum The qualification profile students acquire consists of the following elements: (1) knowledge and comprehension, (2) clinical skills and excellence, (3) communicative competences, (4) therapeutic attitude and (5) occupational competences.

## Methods

The questionnaire “Therapist’s attitude” by Sandell et al. [18] (German version “Therapeutische Haltung (ThAt)” by Klug et al. [19]) v0.3 was used. This instrument measures the attitudes and assumptions of clinicians and psychotherapists in three sections (TASC-2 scales). The first section rates the belief in the curative value of several “ingredients” of physician–patient communication and psychotherapy and consists of 33 items. After conducting principal component analysis Sandell et al. [18] identified 3 scales: adjustment, insight and kindness. The second section and includes 31 items to describe the manner of conducting physician–patient communication or psychotherapy in general. Sandell et al. [18] divided this section into the scales neutrality, supportiveness and self-doubt. The items of both sections are rated on 5‑point Likert-type scales, the first section is ranging from 0 (“does not help at all”) to 4 (“helps a lot”) and the second section from 0 (“do not agree at all”) to 4 (“agree very much”). The third section contains 16 items and intends to rate basic assumption about the nature of physician–patient-communication or psychotherapy and human mind. Therefore continuous bipolar scales are used and the answers are measured by dividing the line into five equal parts. These basic assumptions resulted in three factors: irrationality, artistry and pessimism.

Students of the Medical University of Vienna were asked to fill out the ThAt questionnaires in the fourth year of university prior to the obliged practical workshop “Physician–patient communication with simulated patients” (Gesprächsführungsseminare C) (=t_1_) and after successful participation (=t_2_), i. e. after the final assessment in the end of the 4th year performed in the Objective Structured Clinical Examination (OSCE), again in SP contact.

From the total cohort of students (*n* = 640), a beginning number of *n* = 135 (m:62, f:73) gave informed consent to take part in the study, and 101 (m:54, f:47) could be followed until time point t_2_ after final examination. Students’ mean age was 23.5 years (SD 2.2, min: 20, max: 35 years); 82.3 % stated to have theoretical knowledge concerning psychic functioning, psychiatric illness and treatment options, 16.1 % stated to have personal experience with psychotherapy, 22 % attended an elective course in the psychiatric field. The study was approved by the ethic committee of the Medical University Vienna.

Further, the ThAt questionnaire was analysed content-analytically with 84 items with reference to the Therapeutic Attitudes Scale (TASC-2) [18, 19]. Because of heterogeneity in recent investigations several studies by Sandell et al. [18, 20] were not fully utilizable as for individual clusters/scales a much too high variance was calculated statistically [18–20]. Because of this lack of test-quality criteria here also the content of the ThAt was being processed by Mayring content analysis and new cluster formed [21]. The content analysis was carried out independently of the TASC-2 scales and the evaluation was carried out by two independent raters (interrater reliability *κ* = 0.73).

## Statistics

T-test and U‑test were performed to evaluate a potential significant difference, based on a general significance level of 5 %, between t_1_ and t_2_.

## Results

**Fig. 1:**
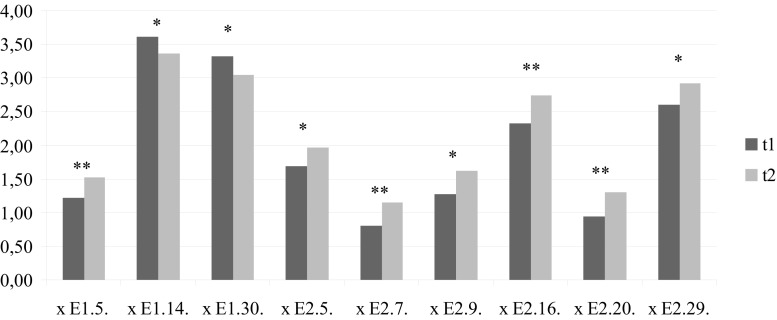
Significant items from section E1 and E2 of the ThAt. * = *p* ≤ 0.05, ** *p* ≤ 0.01, *** = *p* ≤ 0.001; *E1.5.* Helping the patient forget painful experiences, *E1.14.* Helping the patient clarify his/her feelings (this item is included in the TASC-2 scale “Insight”), *E1.30.* Interpreting the patient’s body language (this item is included in the TASC-2 scale “Insight”), *E2.5.* My verbal interventions are brief and concise, *E2.7.* I do not allow long periods of silence during the therapy session, *E2.9.* I often doubt, if my emotions during the session are connected with the patient’s problems or with my own, *E2.16.* I observe my emotions exactly, to recognize what is going on in the patient, *E2.20.* I easily frustrate the patient, *E2.29.* I always communicate the therapeutic goals to the patient in the beginning of a therapy

The scale “Insight” at TASC-2 [x̅(t_1_) = 2.772, x̅(t_2_) = 2.668; *p*(T) = 0.125, *p*(U) = 0.152] and “Irrationality” [x̅(t_1_) = 2.272, x̅(t_2_) = 2.178, *p*(T) = 0.240, *p*(U) = 0.627] decreased slightly. The significant results with regard to the items are shown in Fig. 1. On the item level, the value given by students of item E1.5. “Helping the patient forget painful experiences” increased from t_1_ to t_2_ [x̅(t_1_) = 1.210, x̅(t_2_) = 1.520; *p*(T) = 0.028, *p*(U) = 0.008]. Another item from the “Insight” scale, Item “E1.30. Interpreting the patient’s body language”, decreased [x̅(t_1_) = 3.330, x̅(t_2_) = 3.050; *p*(T) = 0.016, *p*(U) = 0.015].

A significant increase at the scale “targeted approach/self-control” (*p* = 0.000) [16] could be shown. The scale “Self-doubt” showed a tendency to significance (*p*(T) = 0.047, *p*(U) = 0.940); both is shown in Table 1.

**Table 1:** Contrasting juxtaposition of items of the significant TASC-2 scales and clusters developed in the content analytic approach (Mayring content analysis) based on the data of our sample

TASC-2	Cluster after Mayring
*Insight* E1.22. Helping the patient see the connections between his/her problems and childhood E1.28. Working with the patient’s defenses E1.17. Helping the patient understand that old reactions and relations are being repeated with the therapist E1.16. Helping the patient understand that old behaviour and relations are being repeated E1.32. Bringing the patient’s sexuality to the fore E1.33. Helping the patient remember and confront possible sexual abuse E1.18. Supporting the patient in the therapy to reflect on early painful experiences E1.30. Interpreting the patient’s body language E1.19. Giving the patient the opportunity to work with his/her dreams E1.4 Working with the patient’s childhood memories E1.14. Helping the patient clarify his/her feelings E1.31. Letting the patient act out his/her feelings (catharsis)	*Targeted approach/self-control* E2.5. My verbal interventions are brief and concise E2.17. My countertransference is an important instrument in my work E2.29. I always communicate the therapeutic goals to the patient in the beginning of a therapy
	*Uncertainty* E2.7. I do not allow long periods of silence during the therapy session E2.9. I often doubt, if my emotions during the session are connected with the patient’s problems or with my own E2.20. I easily frustrate the patient E2.23. I am often uncertain what to do or say in the session E2.24. I doubt my own ability to contain the patient’s feelings
*Irrationality* F3. By nature, man is rational/irrational F4.1. Human behaviour is governed by free will/by uncontrollable factors F4.2. Human behaviour is governed by external, objective factors/internal, subjective factors F2.6. Psychotherapeutic work is governed by conscious processes/unconscious processes	

Significant gender differences could be found for only two variables: for F4.1 (Human behaviour is governed…by external, objective factors/internal, subjective factors) with 1.94 ± 1.04 for *n* = 119 female and 1.58 ± 1.16 for *N* = 112 male students (*p* = 0.013) and for E2.17 (My countertransference is an important instrument in my work) with 3.32 ± 0.69 for *n* = 120 female and 2.90 ± 0.87 for *n* = 112 male students (*p* = 0.000).

## Discussion

It is important for patients to cope with painful events with psychological mature defence mechanisms (e. g. repression, isolation) to maintain their mental health. In some physically or mentally ill persons immature defence mechanisms (e. g. splitting, devaluation) might be activated due to lack of ability of affect regulation and coping [22]. Students of the fourth year of university learn to observe the different defence mechanisms successfully, as shown in our study, to be able to help patients to develop an adequate coping strategy [23, 24]. Support for this change in students’ attitudes is given due to the increase of values for item “Helping the patient forget painful experiences” from t_1_ to t_2_.

Within the “physician–patient communication C seminars” (Gesprächsführungsseminare C) students are taught and trained among other things to address problems only after having understood, contained and worked through—this processing is also reflected and documented by the attending in form of a reflexion portfolio. This reconsidering procedure could explain the significantly decreased item “Interpreting the patient’s body language”. It can also be seen as an indication of a slight or beginning change from a diagnostic to a rudimentary therapeutic interviewing style. To interpret the body language—after containment and reconsidering the meaning—would be a therapeutic component, exploratory addressing a diagnostic one.

An increase could be shown for the item “My countertransference is an important instrument in my work.” It is important to understand and work with one’s own feelings, which were triggered by the patient, because these can be used for diagnostic purposes [25]. In this item gender differences could be shown, which might reflect either gender-role stereotypes as there were a priori higher values in women, or that female students could achieve this learning objective more easily.

The scales “Insight” and “Irrationality” of TASC-2 decreased slightly. This gives an indication to the effect that medical students after the “physician–patient communication C seminars” concentrate in a more structured way on their objective and lay less emphasis on these parts in the conversation, which correspond to an insight-oriented therapeutic style, e. g. motivational interviewing, clarifying/confronting patients, which is not the primary learning objective of the course.

In the scale “targeted approach/self-control” and in all three items in this category a significant increase of the mean value could be shown. This demonstrates that medical students learn in the workshop to work with more structure and to understand their own feelings better. It remains unclear why the item “I do not allow long periods of silence during the therapy session” increased significantly, even though students were encouraged to allow moments of silence. A potential explanation is a read over the negation word “not”. Another one is the focusing of the diagnostic learning goal [26]. It also has to be mentioned that students are taught to keep pauses especially in the “Gesprächsführungsseminare A and B”, which probably should be recalled in the physician–patient communication with simulated patients—courses in the fourth academic year.

## Limitations

Due to the lack of complete encoding of the ThAt questionnaires no “matched-pairs” comparison could be performed. Further, because of the participation rate of about 20 % of the total cohort, it is not clear if students who participated in the survey are representative or differ from students who declined to take part in the survey. The low participation rate is comparable with other curriculum element evaluations’ return rates and might reflect the voluntariness of evaluation. Further students’ decreasing participation might be due to the fact that after the final examination some already started their holidays, their medical clerkships, clinical electives or started other occupational duties.

Relevant results could be shown with the content analysis of Mayring [21]. Further studies are necessary to revise the ThAt questionnaire and the TASC-2 scales. Due to the significant change in the scale “Insight” and “Irrationality” of TASC-2 (see Fig. 1), we suggest to keep them in the revised version.

## Conclusion

The course “Physician–patient communication with simulated patients” at the medical university improves students’ communication skills. It enables to transfer the just in lectures learnt theoretical knowledge in practice and to train the communicative skills [27]. They learn not only to notice patients’ emotions but also to reflect on them and use them for diagnostic purposes. Furthermore we could show an increased self-reflexivity after participation in the course. Nevertheless, we suggest to implement more lectures and workshops for communicative skills in the curriculum, because taking an adequate history is a crucial skill for diagnosis and therapy success.
